# An Adolescent With a Retropharyngeal Swelling: To Drain or Not to Drain? Keeping a Broad Vision During the COVID-19 Pandemic

**DOI:** 10.1016/j.chest.2022.03.001

**Published:** 2022-08-05

**Authors:** Sybren Robijn, Stan van Keulen, Godelieve Verhage-Damen, Stijn Bekkers

**Affiliations:** a Department of Otorhinolaryngology and Head and Neck Surgery, Radboud University Medical Center, Nijmegen, The Netherlands; b Department of Oral and Maxillofacial Surgery and Oral Pathology, VU University Medical Center/Academic Center for Dentistry Amsterdam (ACTA), Amsterdam, The Netherlands

## Abstract

An 18-year-old patient with a history of COVID-19 (1 month previously) was admitted with malaise and complaints of a stiff neck, a left-sided cervical mass, headache, and difficulty in swallowing and breathing, which had been present for 4 days. Two days after the onset of the first symptoms, a painless skin rash on the legs, arms, palms of both hands, and soles of both feet developed. Despite 2 days of treatment with antibiotics (amoxicillin/clavulanic acid, 500/125 mg three times daily orally), symptoms progressed. On presentation, the patient was alert and oriented, there were no neurologic disorders, and all symptoms related to the recent COVID-19 infection had subsided. His medical history was negative for sexually transmitted diseases, and the patient had received all vaccines except for meningococcus and COVID-19.

## Physical Examination Findings

**Figure 1:**
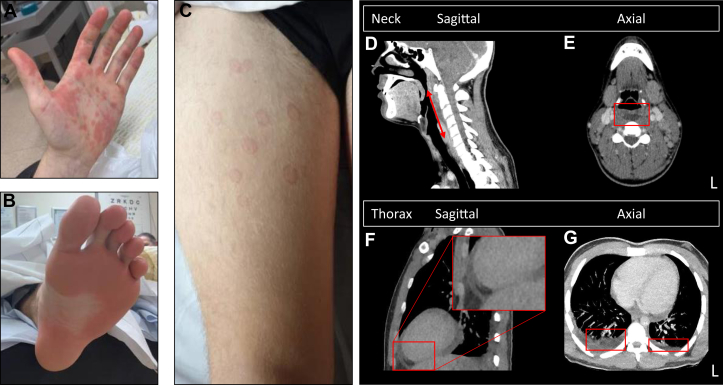
A, Multiple sharply demarcated erythematous papules and plaques on the palmar skin. B, Diffuse erythema of the plantar skin. C, Multiple annular erythematous scaling patches on the proximal extremity. D and E, Sagittal CT scan (D) and axial CT scan (E) of the neck, showing rim-enhanced retropharyngeal fluid collection, 71 mm caudocranial to 11 mm anteroposterior. F, Sagittal CT scan of the chest, showing inferolateral pericardial effusion with a maximum thickness of 12 mm. G, Axial CT scan, showing subtle bilateral pleural effusion.

At presentation, the patient was clinically ill but well oriented and alert, with a body temperature of 40.6 °C, respiratory rate of 22 breaths/min, heart rate of 118 beats/min, BP of 113/71 mm Hg, and oxygen saturation at 100% on room air. Pulmonary examination revealed dyspnea, shallow inspiration, and soft low-pitched breath sounds over the peripheral lung fields. Cardiac examination showed tachycardia with normal first and second heart sounds without murmurs or rubs. There were no neurologic abnormalities. There was stiffness, limited neck rotation, and diffuse and tender swelling over neck levels 2 and 3. There was normal oral mucosa with slight swelling and erythema of the left oral tonsil. Fiber-optic laryngeal endoscopy revealed normal mucosa without swelling. There were multiple sharply demarcated erythematous papules and plaques on the palmar skin, diffuse erythema of the plantar skin, and multiple annular erythematous scaling patches on the proximal extremities (Figs 1A-1C).

## Diagnostic Studies

Although C-reactive protein was elevated (242 mg/L), the leukocyte count was within normal limits. ECG showed diffuse shallow repolarization and a prolonged PQ interval interpreted as a first-degree atrioventricular block. An immediate lumbar puncture showed no signs of meningitis. Contrast-enhanced CT imaging of the neck and thorax revealed an enlarged left tonsil without fluid collection; enlarged mediastinal, retropharyngeal, and left cervical lymph nodes; and a perceived fluid collection (71 mm caudocranial to 11 mm anteroposterior with limited rim enhancement) suggesting a retropharyngeal abscess or fluid collection (Figs 1D, 1E). There were no signs of pulmonary or sinus thrombosis. Subsequently, the patient underwent transcervical exploration of the retropharyngeal region. However, during surgical exploration, no fluid collection or abscess was identified. Cultures of both blood and the retropharyngeal space produced negative results.

Two days postoperatively, the clinical situation deteriorated with chest pain, tachypnea (38 breaths/min), tachycardia (111 beats/min), decreased oxygen saturation (94%), and continued fever (40.1 °C). D-dimer (3,270 ng/mL), troponin (54 ng/L), and N-terminal pro-B-type natriuretic peptide (1,900 pg/mL) were elevated. Follow-up contrast-enhanced CT imaging of the neck and thorax showed pericardial effusion with a maximum thickness of 12 mm (Fig 1F) and subtle bilateral pleural effusion (Fig 1G). Echocardiography confirmed suspected pericarditis with pericardial effusion inferolateral and at the atria. No mediastinal or residual retropharyngeal edema was identified.

*What is the diagnosis?*

*Diagnosis:* Multisystem inflammatory syndrome in children (MIS-C)

## Discussion

This case describes an atypical presentation of MIS-C following infection with SARS-CoV-2. MIS-C was first described in the United Kingdom in the first half of 2020, with the COVID-19 pandemic well over a year underway. Patients presented with a hyperinflammatory syndrome with multiorgan involvement similar to Kawasaki disease. Albeit a rare complication of SARS-CoV-2 infection in children, with an estimated incidence of < 2 per 100,000 patients, these findings countered earlier beliefs that COVID-19 in children mostly passed asymptomatically or with mild symptoms. The more recent literature indicates that MIS-C affects mainly children in the age group of 8 to 10 years, with male children being affected slightly more often (56%). Interestingly, in most cases (70%-80%), patients have no significant comorbidities. Most of the patients require ICU admission, and one-half of the patients present with serious cardiac symptoms such as left ventricular dysfunction, myocarditis, or coronary aneurysms.

The exact pathophysiology of MIC-C is unknown, but it seems to be characterized by an abnormal immune response to COVID-19, and several clinical features seem to, at least partially, mimic Kawasaki disease shock syndrome. Patients generally appear with symptoms 2 to 6 weeks after an acute SARS-CoV-2 infection, in almost all cases presenting with a persistent fever (≥ 3 days) with additional symptoms resulting from involvement of multiple organ systems developing later. Most frequent presenting symptoms include GI symptoms (diarrhea, vomiting, and pain), rash, conjunctivitis, respiratory symptoms, neurologic symptoms (lethargy and confusion), cervical lymphadenopathy, and mucocutaneous and dermatologic findings (swollen lips, strawberry tongue, swollen hands/feet). The more severe and progressive disease mainly presents with signs of shock, Kawasaki-like disease, myocardial dysfunction, respiratory failure, acute kidney failure, serositis, and even encephalopathy.

Diagnostic criteria for MIS-C include age < 21 years, serious illness often leading to hospitalization, fever (> 38.0 °C), laboratory evidence of inflammation, and multisystem involvement in at least two organ systems (cardiac, renal, respiratory, hematologic, GI, dermatologic, or neurologic), recent positive COVID-19 serology, and no plausible alternative diagnosis. The MIS-C specific diagnostic workup should include evaluation of infection parameters, SARS-CoV-2 serology, and, depending on the severity of symptoms, indicators for cardiac, renal, and hepatic function.

Treatment of MIS-C includes IV immunoglobulins often combined with antiinflammatory agents such as steroids and antithrombotic agents. Most patients with MIS-C require critical care. In general, patients with shock should receive fluid resuscitation, inotropic support, and respiratory support when needed. Despite the potential severity of the disease, treatment outcomes are generally favorable, with most children making a full recovery. Mortality has been seen in < 2% of the cases. These patients generally had more comorbidities and tended to be older. Possible sequelae include fatigue, muscle weakness, and, in some reports of patients with cardiac involvement, prolonged depressed cardiac dysfunction. Between 10% and 20% of the patients develop coronary artery aneurysms, so similar to Kawasaki disease that follow-up should include monitoring of cardiac function.

### Clinical Course

The initial differential diagnosis included syphilis, HIV infection, gonorrhea, Kawasaki disease, and MIS-C). Laboratory testing for sexually transmitted diseases produced negative results. Because of the multiorgan involvement, increased infection parameters, negative serology for sexually transmitted diseases, age, and positive SARS-CoV-2 serology, the patient received a diagnosis of MIS-C. After treatment with IV immunoglobulins, colchicine, and acetylsalicylic acid, the clinical situation improved with normalization of body temperature, infection parameters, and disappearance of the dermal abnormalities. Coronary aneurysms were excluded by echocardiography and a coronary CT scan, and the patient was discharged after 6 days of hospitalization. Postoperative outpatient consultations were uneventful except for a little fatigue. Control echocardiography 3 months after discharge revealed no cardiac complications.

Retropharyngeal edema in MIS-C has previously been described; however, we present a case of MIS-C that mimicked a retropharyngeal phlegmon, thereby delaying the diagnostic process. In retrospect, surgery and IV antibiotics were unnecessary, and earlier IV immunoglobulins could have prevented further deterioration. Being acquainted with cues such as dermal abnormalities and multiorgan involvement after a recent history of COVID-19 in children or adolescents could guide physicians in diagnosing MIS-C.

## Clinical Pearls

1. *MIS-C is a rare complication of COVID-19 that occurs predominantly in healthy children and adolescents 2 to 6 weeks after an acute SARS-CoV-2 infection and often requires admission to the critical care unit.*
2. *Symptoms of MIS-C are diverse and include rash, conjunctivitis, GI, respiratory, cardiac, and neurologic symptoms, but physicians should also be aware of an atypical presentation, such as retropharyngeal edema.*
3. *MIS-C occurs mainly in children who have COVID-19 asymptomatically or with only limited symptoms, so MIS-C should be in the differential diagnosis for* *children and adolescents with* *critical* *ill* *ness* *during the COVID-19 pandemic.*
4. *Treatment of MIS-C includes IV immunoglobulins often combined with antiinflammatory agents such as steroids and antithrombotic agents.*
